# Clinical Cases

**Published:** 1890-05

**Authors:** G. W. Sherbino

**Affiliations:** Abilene, Texas


### CLINICAL CASES.

G. W. SHERBINO, M. D., ABILENE, TEXAS.

CEPHALALGIA.—Reading some time ago in THE HOMŒOPATHIC PHYSICIAN of a case cured by Sepia, in having the sensation as though the neck were swollen or enlarged, I am led to report this case.

A lawyer's wife had suffered with headache and pain in the shoulders and side. There was nothing peculiar about the pain, but she said her neck was swelled. It felt that way, and she kept feeling her neck all the time I was in the house. I could see no enlargement. The pain was worse at night after lying down for awhile. It became worse in the morning when commencing to move about. As she was engaged about her work she was better. She always noticed it more when she was quiet. "She had no faith," but Rhus-tox.$^{cm}$, two doses, cured her, faith and all, and she has remained well ever since.

METRORRHAGIA.—A married lady had been flooding for three weeks from a miscarriage. She said: "At night I am troubled so about a peculiar feeling I have. I try to go to sleep, and I feel all the time as though I was '*standing on my head,*' or *that my feet and legs were in the bed, and my head was down on the floor.*" She had other symptoms. Baptisia, two doses of which relieved.

CONCUSSION OF THE BRAIN.—A man living twenty-five miles in the country was taken suddenly with unconsciousness. He does not remember getting out of bed, or eating breakfast. Left the table and went out to a wagon. He leaned his head upon a trunk-rack for a moment, then suddenly straightened himself up, and fell backward in a perfectly rigid state, striking his head upon the hard ground. He came to his senses about noon, and was brought home. I saw him in the evening. He complained of terrible pain throughout the back of the head, and all through the brain, of a bruised character. I gave him Arnica $^{cm}$, one dose, and nothing else. The next morning the pain was all gone from the back of the head. He was ever so much better, only had slight pain in the frontal region. Next day entirely well. I have no reason for reporting the case further than for the potency used. One dose did the work. I want to put it on record for the benefit of the weak, and not let it die an ignominious death in the dark.

POST-PARTUM HEMORRHAGE.—Mrs. W. J., æt. eighteen, primipara, was taken with labor in the morning; gave birth to male child. Breech presentation. Labor lasted about twelve hours ; the second stage three hours. Very hard labor. I used pressure of the abdomen with each pain, which helped her very much. I had no trouble to remove the after-birth. Soon afterward she said "I am flooding." I put my hand on the abdomen and found the uterus relaxed up as high as the umbilicus. I grasped the uterus with my hand and soon firm contraction came on, but in a few moments relaxation would take place. I gave Bell., as I thought it the remedy, but no relief. She thought the pain went from her back through to uterus or pubes. I gave Sabina, but it acted no better than Bell. Now she says, "Doctor, whenever a pain comes on I feel as if I wanted to get up ; my bowels feel as if they wanted to move." I gave Nux-vom.$^{cm}$, one dose. I needed no more pressure over the uterus, as she said, "I feel better," and the hemorrhage was soon controlled. From this case we get the following deductions :

1st. The impossibility of curing with a remedy not indicated.

2d. There was no need of a hypodermic injection of fluid extract of Ergot.

3d. There was no need of dashing cold water over the abdomen, or packing the uterus with ice.

4th. She was cured pleasantly, safely, and speedily, according to Hahnemann's instructions.

Acne Facialis.—Mr. ——, æt. twenty, has had pimples on his face, especially the nose, too horrible to look at. Has had this for three years. Asked me several times to cure him. I told him I made no pretensions to curing pimples. The only symptom he had was profuse foot-sweat, fetid odor, cold and clammy in the winter. Sanicula$^{50\,m}$ (Sk.), two doses, cured in one month, so that his face is perfectly smooth.

Cephalalgia.—Headache for two days. She says the pain commenced in the region of the supra-orbital nerve, passing back along the base of the brain and down her neck. The pain was all through her head. Eyes and lids were very red and swollen, and the pain was terrible ; worse from motion or moving the eyes. Actea-rac.$^{40\,m}$, one dose, cured. The pain in the region of the supra-orbital nerve is characteristic of this remedy.

Cephalalgia.—Baptisia$^{dmm}$ (Swan's).—Mrs. D., æt. thirty-five, has had headache for years. It comes once a month, and sometimes every week, lasts for several days. She took treatment from a "homœopath," who said that no medicine would ever do her any good. After a long search I obtained the following: She gets sleepy and stupid as the headache comes on, her hands are dead and no feeling in them. A loss of sensibility. Pillow feels hard. Cannot find an easy place to lay her head. Bed is so hard she cannot lie long in one place. Baptisia$^{dmm}$ (Swan). One dose relieved for three weeks. One dose at the commencement always relieves in a short time. She is now in better health than for years.

